# Plant-derived bioactives and nutraceuticals in chronic prostatitis/chronic pelvic pain syndrome: clinical evidence, pain-sensitization mechanisms and nutritional translation

**DOI:** 10.3389/fnut.2026.1966858

**Published:** 2026-10-20

**Authors:** Xue Liu, Junfu Mo, Zhirui Sun, Min Liu, Zhaoxu Yang

**Affiliations:** 1 Department of Andrology, Jiangsu Province Hospital of Chinese Medicine, Affiliated Hospital of Nanjing University of Chinese Medicine, Nanjing, China; 2 Chengdu University of Traditional Chinese Medicine, Chengdu, China; 3 The First Affiliated Hospital of Guangzhou University of Chinese Medicine, Chongqing Hospital (Chongqing Beibei District Traditional Chinese Medicine Hospital), Chongqing, China

**Keywords:** botanical extracts, chronic prostatitis/chronic pelvic pain syndrome, dietary polyphenols, nutraceuticals, nutritional translation, oxidative stress, pain sensitization, plant-derived bioactives

## Abstract

Chronic prostatitis/chronic pelvic pain syndrome (CP/CPPS) is a common cause of persistent pelvic pain in men, often accompanied by urinary, sexual and psychosocial symptoms. Clinical heterogeneity and inconsistent treatment responses have shifted mechanistic attention beyond the prostate to peripheral nociceptor sensitization, spinal amplification and altered central pain processing. Plant foods and botanical materials contain polyphenols, terpenes, carotenoids, alkaloids and isothiocyanates that influence inflammatory, redox, nociceptive and microbiota-related pathways. CP/CPPS trials have evaluated these exposures mainly as standardized extracts, concentrated supplements or fixed nutraceutical formulations. This targeted narrative review integrates their clinical evidence with pain-sensitization biology and the determinants of nutritional exposure. Literature searches covered PubMed, Web of Science Core Collection and Scopus for publications dated on or before 8 August 2026, with priority given to controlled and prospective human CP/CPPS studies. Selected standardized extracts have shown clinical benefits, while quercetin is supported by preliminary clinical evidence from one small placebo-controlled trial. Curcumin and antioxidant products have been tested mainly as multi-ingredient formulations. β-Caryophyllene, berberine and sulforaphane provide emerging experimental or adjacent-condition evidence. Across candidates, the most consistent biological links involve peripheral inflammation, oxidative stress, nociceptor excitability and lower urinary tract function. Together, these findings support a model in which plant-derived bioactives may reduce peripheral inputs that sustain pain sensitization. Formulation, bioavailability and safety are central to translating these findings into nutritional interventions. Larger randomized trials should evaluate clinical benefits and test the proposed links to pain sensitization.

## 1 Introduction

Chronic prostatitis/chronic pelvic pain syndrome (CP/CPPS) is classified as National Institutes of Health category III prostatitis. It is the predominant chronic prostatitis phenotype and an important cause of persistent pelvic pain in men (1, 2). Depending on the population and case definition, prostatitis-like symptoms affect approximately 2 to 10% of adult men (3, 4). Pelvic, perineal, genital or ejaculatory pain may coexist with lower urinary tract symptoms, sexual dysfunction, psychological distress and impaired quality of life, despite no demonstrable bacterial infection (2, 5, 6). Antibiotics, α-adrenergic blockers, anti-inflammatory agents, neuromodulatory drugs and pelvic-floor interventions are widely used, but responses remain inconsistent (7–9). This heterogeneity points beyond a single prostatic lesion or inflammatory process.

Current pathophysiological models extend beyond the prostate. Local inflammation and oxidative stress may initiate or amplify nociceptive signaling in some patients, but inflammatory findings do not consistently track the severity or persistence of pain. Peripheral nociceptor sensitization, neurogenic inflammation, pelvic-floor dysfunction and viscerosomatic convergence can maintain afferent input. Spinal neuroimmune activation and altered supraspinal pain processing may then contribute to chronicity in susceptible individuals (10–13). Autonomic imbalance, stress-related neuroendocrine regulation, psychological factors and microbiota-related signaling further shape symptom expression (14–17). These processes interact and vary among patients. This variability underlies the clinical heterogeneity represented by UPOINT: urinary, psychosocial, organ-specific, infection, neurologic/systemic and tenderness domains (18).

Plant foods and botanical materials supply polyphenols, terpenes, carotenoids, alkaloids and other bioactive constituents. Their reported activities intersect with inflammatory and redox signaling, mast-cell activation, nociceptor excitability, neuroimmune communication and microbial metabolism. CP/CPPS intervention studies have mainly tested concentrated exposures, including standardized extracts, single-compound supplements and multi-ingredient nutraceuticals. Selected preparations have improved pain or multidomain symptom scores (7, 19). Interpretation depends on source, food matrix, formulation, dose, route and bioavailability (20–23). Ordinary diet and concentrated products represent distinct exposures (19, 24). Recent reviews have examined polyphenols and isothiocyanates across genitourinary diseases and the neuroendocrine mechanisms of CP/CPPS (25, 26). Building on these complementary perspectives, a focused synthesis is needed to relate the clinical evidence for plant-derived interventions in CP/CPPS to pain-sensitization mechanisms and the factors that shape their nutritional translation.

This narrative review integrates clinical evidence for plant-derived bioactives and nutraceuticals in CP/CPPS with mechanistic insights into peripheral, spinal and supraspinal pain sensitization. It examines how dietary context, formulation, bioavailability and safety influence the translation of these findings into nutritional interventions (Figure 1).

**Figure 1:**
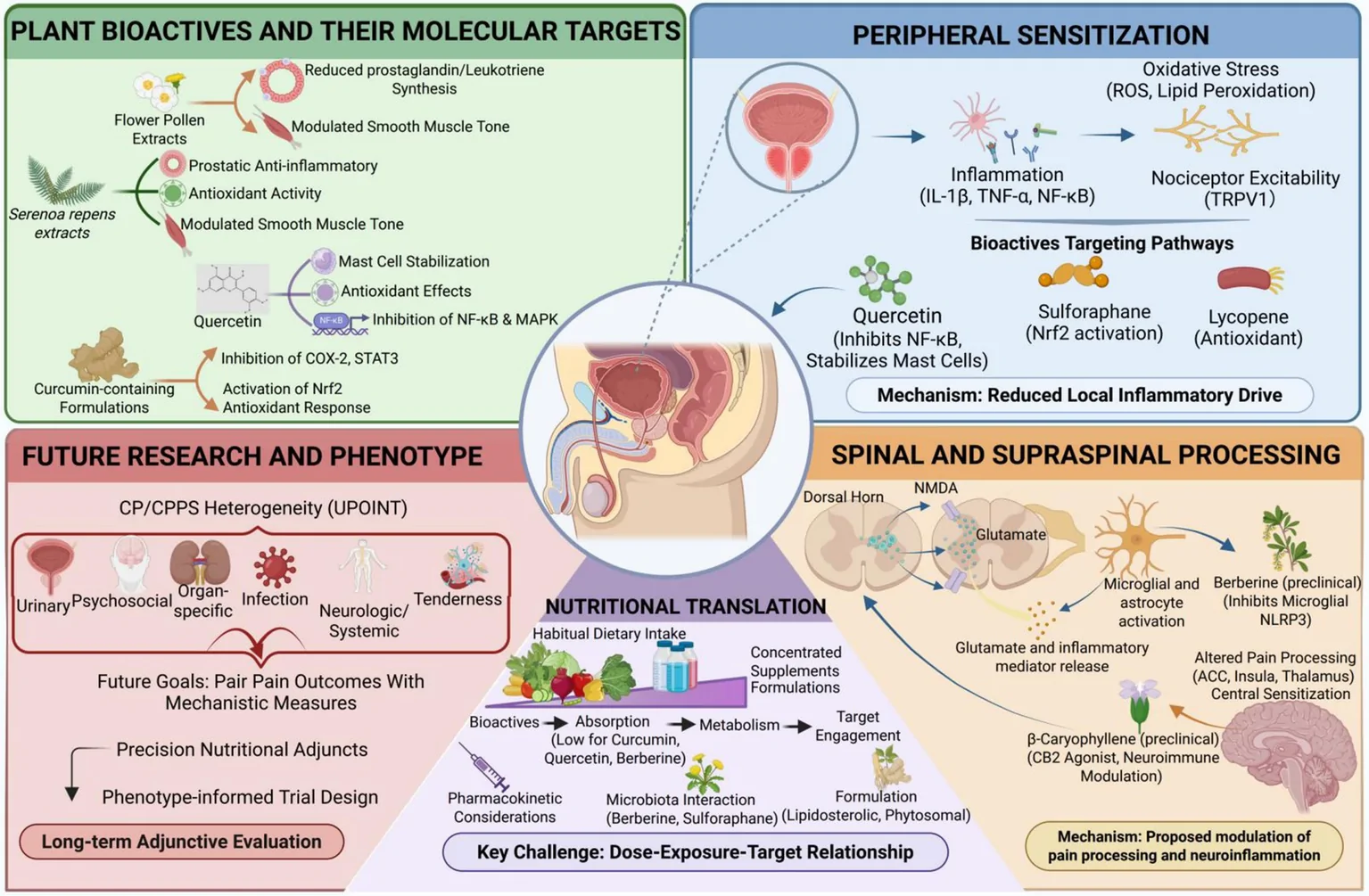
Framework linking plant-derived bioactives to pain-sensitization biology in CP/CPPS: reported molecular targets of the main preparations (green), peripheral inflammatory and redox processes (blue), spinal and supraspinal processing (orange), UPOINT phenotype domains and research priorities (red), and translation from dietary intake and concentrated preparations to target engagement (purple). β-Caryophyllene, berberine and sulforaphane are discussed as preclinical candidates (the figure was created with BioRender).

## 2 Methods

This narrative review was last updated to cover literature published on or before 8 August 2026. PubMed, Web of Science Core Collection and Scopus were searched using two concept groups: CP/CPPS and plant-derived exposures (bioactives, phytotherapy, nutraceuticals and diet). Synonyms were combined with OR and concept groups with AND. Searches used MeSH and title/abstract terms in PubMed, Topic fields in Web of Science, and title/abstract/keyword fields in Scopus, without language or study-design filters. During manuscript revision, the documented searches retrieved 5,270 raw records (1,530 from PubMed, 999 from Web of Science and 2,741 from Scopus). Conservative automated deduplication left 2,992 candidate records before final publication-date and relevance assessment.

Selection was purposive, based on relevance to CP/CPPS and the clinical or mechanistic question. Controlled and prospective human CP/CPPS studies were prioritized. Observational dietary studies and human or experimental mechanistic studies, including those in related pain conditions, provided context. Targeted mechanistic and safety searches and reference-list checking complemented these searches. Findings were interpreted by preparation, dose, route and evidence directness. Complex herbal formulae lacking reproducible compositional information were excluded.

This review aimed to integrate clinical evidence and mechanistic findings from a nutritional perspective. Given heterogeneity in populations, preparations, doses and outcomes, findings were synthesized narratively without quantitative pooling.

## 3 Biological basis of persistent pain in CP/CPPS

The inflammation-sensitization axis is an organizing framework whose relevance varies by phenotype. Local inflammatory and redox signals can increase nociceptor excitability and sustain pelvic afferent input. Persistent input may contribute to spinal amplification and altered supraspinal processing in susceptible individuals (11, 12, 27). Direct human CP/CPPS studies document peripheral inflammatory abnormalities in selected cohorts (28–31), and phenotyping studies report features compatible with altered central pain processing (32–35). This multilevel framework helps interpret the clinical and experimental evidence on plant-derived interventions in CP/CPPS (Figure 2).

**Figure 2:**
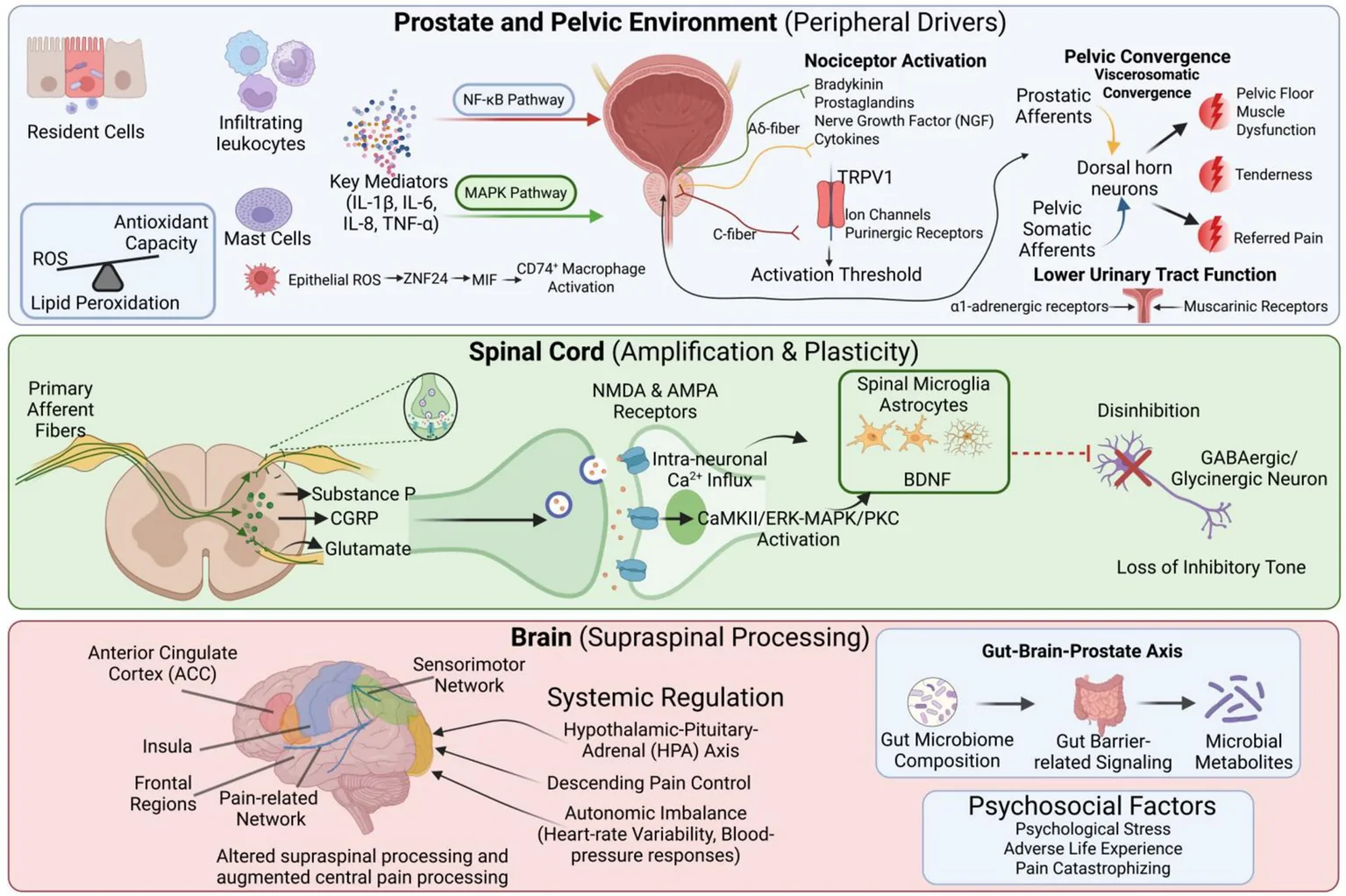
Multilevel processes that may sustain pain in CP/CPPS: peripheral drivers in the prostatic and pelvic environment (top), spinal amplification and loss of inhibitory tone (middle), and supraspinal processing with systemic and psychosocial regulation (bottom). CGRP, calcitonin gene-related peptide; NGF, nerve growth factor (the figure was created with BioRender).

### 3.1 Local inflammation, oxidative stress and peripheral sensitization

Local inflammatory activity is detectable in a subset of men with CP/CPPS, particularly those meeting inflammatory category IIIA criteria. Its magnitude varies and may not track pain severity. Elevated cytokines and chemokines have been reported in expressed prostatic secretions, seminal plasma and post-massage urine. Commonly studied mediators include interleukin-1 beta (IL-1β), IL-6, IL-8 and tumor necrosis factor-alpha (TNF-α) (28–31). Resident prostatic cells, infiltrating leukocytes and mast cells may contribute to this inflammatory milieu. Experimental autoimmune prostatitis models also support autoimmune and mast-cell-mediated pathways (10, 36). These findings identify inflammatory subphenotypes in which local immune and redox modulation is biologically relevant. Reactive oxygen and nitrogen species accumulate when oxidative stress exceeds endogenous antioxidant defenses. Some inflammatory prostatitis cohorts, including leukocytospermic cases, show increased seminal lipid-peroxidation markers and reduced antioxidant capacity (37, 38). These changes may amplify cytokine release through redox-sensitive transcription. Nuclear factor-kappa B (NF-κB) and mitogen-activated protein kinase (MAPK) cascades coordinate inflammatory mediator transcription. The cyclooxygenase-2/prostaglandin E2 pathway propagates inflammatory pain signaling, whereas the Kelch-like ECH-associated protein 1 (Keap1)/nuclear factor erythroid 2-related factor 2 (Nrf2) pathway governs counter-regulatory antioxidant responses (39–41).

These transcriptional and redox hubs form a peripheral convergence point for several plant-derived bioactives. Local inflammation may initiate symptoms, coexist with other drivers, or arise downstream of pelvic-floor, autonomic or stress-related processes. A bidirectional model therefore accommodates several routes into persistent pain. A 2026 multimodal study in chronic nonbacterial prostatitis identified an epithelial ROS-ZNF24-macrophage migration inhibitory factor pathway linked to CD74-positive macrophage immunometabolic activation (42). The combined plasma, transcriptomic, cellular and animal data define a candidate epithelial-immune node for clinical validation and mechanism-focused nutritional research. Inflammatory mediators also excite and sensitize the primary afferent nociceptors that innervate the prostate and pelvic structures. Bradykinin, prostaglandins, nerve growth factor (NGF) and pro-inflammatory cytokines can lower the activation threshold of unmyelinated C-fibers and thinly myelinated Aδ-fibers, converting a transient insult into persistent afferent drive (13, 43). Sensitization can also recruit normally quiescent “silent” visceral nociceptors; once activated, these fibers become mechanosensitive and spontaneously active, promoting the transition from acute nociception to persistent visceral hypersensitivity. Experimental autoimmune prostatitis has directly implicated transient receptor potential vanilloid 1 (TRPV1), while broader purinergic and ion-channel mechanisms remain part of the proposed sensory model (13, 44).

Activated nociceptors release calcitonin gene-related peptide (CGRP) and substance P into the periphery. The resulting neurogenic inflammation promotes vasodilation, vascular permeability and further immune-cell recruitment. This feed-forward neuroimmune loop offers one explanation for pain that persists after the initiating inflammatory stimulus has waned. Neurotrophins, including NGF and brain-derived neurotrophic factor (BDNF), may also translate sustained peripheral activity into synaptic and transcriptional changes associated with central plasticity (11, 13, 43). Viscerosomatic convergence gives peripheral sensitization a clinical footprint. Pelvic visceral and somatic afferents converge on shared dorsal horn neurons, allowing prostatic sensitization to present as pelvic-floor hypertonicity, myofascial trigger points and referred pain (45–48). Pelvic-floor dysfunction can then amplify visceral symptoms and contribute to overlapping pain, urinary and muscular complaints (47–50). This convergence provides an anatomical basis for the tenderness and neurologic/systemic features observed in CP/CPPS.

### 3.2 Spinal amplification and altered central pain processing

Persistent high-frequency nociceptive input can produce central sensitization in experimental dorsal-horn systems. Glutamate, substance P and CGRP promote postsynaptic depolarization and N-methyl-D-aspartate receptor activation. Downstream calcium signaling engages protein kinase C, calcium/calmodulin-dependent kinase II, extracellular signal-regulated kinase and p38 MAPK pathways. These cascades increase excitatory transmission and lower activation thresholds, providing a model for interpreting possible spinal amplification in CP/CPPS (27, 51). Loss of gamma-aminobutyric acid-mediated and glycinergic inhibitory tone can further amplify dorsal horn output. Activated spinal microglia and astrocytes may release IL-1β, TNF-α and BDNF, sustaining glia–neuron feedback loops after peripheral input declines (11, 51).

Human studies support altered central pain processing in a subset of patients. CP/CPPS and broader urological chronic pelvic pain cohorts show altered non-pelvic pressure-pain sensitivity and electrophysiological findings compatible with central sensitization (32–34). Neuroimaging studies separately report differences in pain-related and sensorimotor brain networks (12, 35, 52–54). A 2025 resting-state functional MRI study of 45 men with CP/CPPS and 45 matched controls found symptom-related differences in the anterior cingulate cortex, insula, thalamus and frontal regions (55). Together, these findings define a disease-level central phenotype and identify candidate longitudinal endpoints for treatment studies. Central sensitization is a neurophysiological construct. Nociplastic pain describes pain arising from altered nociception that is not fully explained by nociceptive or neuropathic mechanisms; the terms are related but not synonymous (56). Questionnaire scores, widespread pressure sensitivity and neuroimaging can characterize augmented central processing, while cellular central sensitization requires convergent physiological evidence (12, 33, 57). The available data support centrally amplified or nociplastic features in a clinically relevant subset of patients. Differences in phenotype, cohort composition and sensory-testing methods help explain variation in widespread hyperalgesia (33, 57). This heterogeneity supports phenotype enrichment and multimodal assessment in future trials.

### 3.3 Systemic regulation and phenotype heterogeneity

The maintenance of CP/CPPS reflects bidirectional integration among neural, immune, endocrine and psychological systems. Psychological stress, adverse life experience and pain catastrophizing may modulate descending pain control and the hypothalamic–pituitary–adrenal axis, thereby contributing to altered pain modulation or central amplification and greater symptom severity (15, 16, 58–60). Chronic pain and psychological distress may also become mutually reinforcing in these patients. Autonomic and neuroendocrine pathways provide further links between pelvic symptoms and systemic regulation. Case–control studies have reported abnormalities in heart-rate variability, blood-pressure responses and sympathetic skin responses in some men with CP/CPPS (13, 14, 61). Substance P and CGRP may also promote neurogenic inflammation. Altered hypothalamic–pituitary–adrenal-axis measures have been described, although their direction and clinical meaning vary (15, 61). These observations support a neuro-endocrine-immune network.

The proposed gut-brain-prostate axis adds another systemic layer. Small observational studies report differences in gut microbial composition between men with CP/CPPS and controls (17, 62–64). Microbial metabolites and immune signaling could influence systemic and neural regulation of pelvic pain. These findings provide an initial basis for microbiota-active nutritional research that pairs microbial function with host and symptom outcomes. Viscerosomatic convergence is most relevant to pelvic referred pain and pelvic-floor symptoms. Overlap with irritable bowel syndrome and other chronic pain conditions may reflect shared abnormalities in pain modulation, autonomic regulation and symptom amplification. Clinically, this systems biology appears as phenotype heterogeneity. UPOINT does not define separate diseases; it provides a pragmatic map of the domains most likely to dominate in a given patient (18, 65). This bridge from mechanism to phenotype informs the phenotype-aware interpretation developed in Section 5.

## 4 Plant-derived bioactives and nutraceuticals in CP/CPPS

Dietary context and formulation shape the exposure achievable with each intervention. The following sections move from clinically studied preparations to emerging compounds, distinguishing human CP/CPPS findings from experimental mechanisms and evidence from other conditions (Table 1).

**Table 1:** Summary of clinical evidence for plant-derived active substances in CP/CPPS.

Plant active substances/extracts	Basis of clinical evidence	Key findings	Proposed mechanisms and evidence context
Flower pollen extracts (e.g., *Cernilton*)	Placebo-controlled trials of Cernilton (139 men) and Prostat/Poltit (60 men), and comparative studies.	1. A placebo-controlled phase III trial in 139 men with category IIIA CP/CPPS reported improved NIH-CPSI pain, quality-of-life and total scores at 12 weeks. A separate trial of Prostat/Poltit in 60 men reported greater symptomatic improvement than placebo at 6 months. 2. A pollen-plus-vitamin product improved symptoms and reduced seminal IL-8 compared with bromelain.	Experimental evidence: reduced prostaglandin/leukotriene synthesis and regulation of urethral smooth-muscle tone.
Saw Palmetto (*Serenoa repens*)	Placebo-controlled trial (226 randomized; 221 analyzed) and comparative studies.	1. A defined *Serenoa repens* extract improved NIH-CPSI total and domain scores versus placebo at 12 weeks. 2. In category IIIA disease, *Serenoa repens* plus selenium and lycopene improved NIH-CPSI and urinary measures more than *Serenoa repens* alone.	Experimental evidence: interactions with α1-adrenergic and muscarinic receptors in the lower urinary tract.
Quercetin	One small placebo-controlled trial (30 randomized; 28 completed); preliminary, short-term evidence.	1. A small placebo-controlled trial (30 randomized; 28 completed) reported at least 25% symptom improvement in 67% with quercetin versus 20% with placebo after 1 month. 2. A separate open-label study evaluated quercetin with bromelain and papain.	Anti-inflammatory, antioxidant, mast cell stabilization. Experimental models show inhibition of NF-κB signaling, with potential to regulate central neuroimmunity.
Curcumin-containing formulations	Placebo-controlled rectal-combination trial (60 enrolled; 48 completed) and uncontrolled oral-combination study (20 men).	1. Rectal curcumin plus Calendula improved NIH-CPSI and related outcomes in a placebo-controlled trial (60 enrolled; 48 completed). 2. Oral curcumin, quercetin, hyaluronic acid and chondroitin sulfate improved NIH-CPSI pain and urinary-domain scores at 30 days in an uncontrolled study of 20 men; these changes were not significant at 90 days.	Experimental studies implicate NF-κB and STAT3 signaling. Oral bioavailability is formulation-dependent.
Multi-ingredient antioxidant formulations	Untreated-control comparison and a randomized add-on trial (47 men) of different combinations.	A formulation containing lycopene, EGCG, ellagic acid, selenium and zinc was associated with reduced seminal leukocytes and improved NIH-CPSI pain scores in inflammatory category IIIA CP/CPPS compared with an untreated control group.	Constituents act through distinct antioxidant and immune pathways.
β-Caryophyllene	Preclinical evidence; no human CP/CPPS intervention trial identified.	Reduced inflammatory signaling and pain-like behavior in mouse neuropathic-pain models; activity also reported in experimental interstitial cystitis.	CB2 receptor agonism and neuroimmune signaling without CB1-mediated psychotropic effects.
Berberine	Preclinical evidence; no human CP/CPPS intervention trial identified.	Reduced prostatic inflammation and oxidative stress in rats with adjuvant-induced prostatitis.	May act via the “Gut-Brain-Prostate” axis, regulating gut microbiota and metabolites.
Sulforaphane	Preclinical evidence; no human CP/CPPS intervention trial identified.	Reduced inflammatory infiltration and mechanical pain responses in mice with experimental autoimmune prostatitis.	Activates Nrf2 pathway, inhibits NLRP3 inflammasome, targeting peripheral redox-inflammatory nodes.

Clinical evidence summaries consider study design, study-specific sample sizes, consistency and directness to human CP/CPPS. Proposed mechanisms are supported mainly by preclinical evidence.

Dietary patterns provide the background against which concentrated botanical preparations are consumed and may act. Whole foods deliver repeated low-dose mixtures within matrices containing fiber, fats, micronutrients and other phytochemicals. Standardized extracts differ in dose, composition and reproducibility. Whole-diet intervention evidence in CP/CPPS is still at an early stage. Baseline diet should therefore be recorded as a potential effect modifier because it may influence symptoms, adherence, inflammatory tone and microbial metabolism. In one questionnaire study, men with CP/CPPS associated symptom aggravation with spicy foods, coffee, alcohol, tea or chili. Some participants perceived improvement with selected beverages or bowel-regulating products (66). These self-reported patterns identified candidate dietary triggers and support prospective recording of habitual diet, major dietary changes and food-supplement co-exposures. More recently, a 2026 study of 313 men used food-frequency data to derive dietary patterns and identified a red-meat-and-processed-food pattern as the dietary feature most strongly associated with CP/CPPS symptom severity (67). These findings extend earlier reports of individual food triggers and support incorporating habitual dietary context into future phenotype-informed nutritional studies.

### 4.1 Clinically studied botanical extracts and food-derived compounds

#### 4.1.1 Flower pollen extracts

Preparations made from *Secale cereale*, *Phleum pratense* and *Zea mays* pollen are standardized to fractions such as Cernitin T60 and GBX (68–70). They are among the most extensively studied botanical preparations in CP/CPPS. Experimental work suggests peripheral actions: reduced prostaglandin and leukotriene synthesis, attenuation of local inflammatory signaling and modulation of urethral smooth-muscle tone.

A placebo-controlled trial of a standardized pollen preparation in men with inflammatory category IIIA CP/CPPS reported improvements in NIH Chronic Prostatitis Symptom Index (NIH-CPSI) pain, quality-of-life and total scores (71). Comparative and prospective studies also suggest lower symptom burden, with changes in seminal IL-8 indicating a possible link to local inflammatory activity (72–74). Pooled estimates vary with preparation, comparator and study quality (75).

The strongest individual study was a multicenter phase 3 trial in 139 men with category IIIA CP/CPPS (71). Participants received Cernilton or placebo for 12 weeks, with change in the NIH-CPSI pain domain as the prespecified primary endpoint. Pain, quality-of-life and total scores improved more with Cernilton. A response was defined as a reduction of at least six points or 25% in total NIH-CPSI. This threshold was met by 70.6% of treated participants and 50.0% of placebo recipients. A separate six-month randomized, double-blind, placebo-controlled trial in 60 men found greater symptomatic improvement with Prostat/Poltit, a different pollen preparation, than with placebo (76).

Active-comparator trials help distinguish the symptom domains that respond. In a randomized study of 100 men, pollen extract and Eviprostat produced similar eight-week changes in NIH-CPSI domains (72). Another 100-patient study examined add-on treatment for persistent symptoms during α1-blocker therapy. Cernitin improved pain more than tadalafil, whereas tadalafil improved lower urinary tract symptoms more (73). Such differences may be obscured by the total NIH-CPSI score. Pollen extract plus vitamins also produced greater symptom improvement and lower seminal IL-8 than bromelain (74). These findings concern the combination, with lower IL-8 consistent with reduced peripheral inflammatory activity.

Clinical studies have also examined combinations and alternative routes. In a 26-week randomized, single-blind phase III study, 124 men received a short course of suppositories or oral ibuprofen. The suppositories contained pollen extract, hyaluronic acid and vitamins and produced more persistent improvements in symptoms and quality of life (77). Other prospective or randomized comparisons favored a pollen-plus-vitamin product over a defined *Serenoa repens* extract or quercetin-containing bioflavonoids. A 2025 multicenter prospective study reported greater symptom reductions with pollen extract, alone or with an α-blocker, than with an α-blocker alone (78–80).

#### 4.1.2 Saw palmetto (*Serenoa repens*)

Its fruit yields a lipidic extract containing fatty acids, phytosterols and other minor constituents (22). Controlled studies in men with CP/CPPS have reported reductions in NIH-CPSI total and domain scores with defined extracts (81, 82). Comparative and add-on studies also suggest benefit during standard medical treatment (83, 84). Lipid profiles and extraction methods differ substantially among commercial products (22).

The largest placebo-controlled trial of a defined *Serenoa repens* extract in CP/CPPS was conducted at 11 centers. It randomized 226 participants 2:1 to a defined extract or placebo for 12 weeks. The intention-to-treat analysis included 221 participants (148 and 73, respectively). Change in total NIH-CPSI was the prespecified primary endpoint; improvement was greater with the extract at week 12. A reduction of at least six points, the secondary responder endpoint, occurred in 73.0% versus 32.9% of participants (*p* < 0.0001) (81). An earlier one-year open-label comparison in 64 men found little change in NIH-CPSI with saw palmetto, while several outcomes improved with finasteride (83). Differences in extract composition, comparator and study design may help explain the contrasting findings.

Combination studies have examined *Serenoa repens* with other drugs or nutritional ingredients. In category IIIA CP/CPPS, *Serenoa repens* combined with selenium and lycopene improved NIH-CPSI and urinary measures more than *Serenoa repens* alone, and reduced leukocyte counts (82). A seven-arm randomized study enrolled 350 young men with CP/CPPS and subfertility. It compared tadalafil, L-carnitine and *Serenoa repens* alone and in combination (85). The triple regimen produced the largest three-month improvements in NIH-CPSI, urinary and semen outcomes.

Experimental studies suggest effects on prostatic inflammatory and redox pathways and lower urinary tract smooth-muscle tone. *In vitro* and ex vivo experiments in the rat lower urinary tract show interactions with α1-adrenergic and muscarinic receptors (86). An ex vivo study of rat prostate tissue also found reduced inflammatory signaling with a mixture of *Serenoa repens*, *Crocus sativus* and *Pinus massoniana* extracts (87).

Products sold under the same botanical name can differ in fatty-acid, phytosterol and minor-constituent profiles because of source material, extraction solvent and manufacturing process (22).

#### 4.1.3 Quercetin

This dietary flavonol occurs in onions, apples, berries, capers and tea, but concentrated supplements produce exposures distinct from ordinary dietary intake. An early placebo-controlled trial in men with category III CP/CPPS reported greater symptom improvement with quercetin than with placebo (88). The trial randomized 30 men to quercetin 500 mg twice daily or placebo for 1 month; 28 completed the blinded comparison (88). Mean symptom scores fell from 21.0 to 13.1 with quercetin and from 20.2 to 18.8 with placebo. At least 25% improvement occurred in 67% versus 20% of participants. This small trial provides preliminary clinical evidence of short-term symptom improvement with quercetin. A separate open-label study examined quercetin combined with bromelain and papain.

In experimental systems, quercetin shows anti-inflammatory, antioxidant and mast-cell-stabilizing activity. It can modulate cytokine production, redox-sensitive transcription and nociceptor-related signaling. In a rat model of chronic prostatitis, it reduces prostatic inflammatory cytokines, strengthens antioxidant defenses and inhibits NF-κB and MAPK signaling (89). Preclinical studies outside CP/CPPS report reduced P2X4 expression and p38 MAPK activation in dorsal-root-ganglion satellite glia. They also describe AMPK/MAPK-dependent effects after chronic constriction injury and modulation of microglial NLRP3-mitophagy crosstalk (90–92).

The CP/CPPS trial reports short-term symptom improvement at supplement-level dosing. Prostatitis models and neuroimmune studies identify peripheral inflammation and glial signaling as candidate pathways linking quercetin to pain sensitization (88–90, 92).

### 4.2 Formulation-based nutraceutical evidence

#### 4.2.1 Curcumin-containing formulations

Curcumin is a dietary polyphenol derived from turmeric. Human CP/CPPS studies in this section have mainly tested multi-ingredient products, so the clinical findings concern the combinations as a whole. A rectal curcumin-plus-Calendula product improved NIH-CPSI and related outcomes (93). The randomized phase II study enrolled 60 men; 48 completed a single-blind, placebo-controlled comparison of curcumin and Calendula suppositories (93). NIH-CPSI, visual analog pain scores, urinary flow and sexual-function measures improved.

A 2025 uncontrolled prospective study tested an oral combination in 20 men with CP/CPPS. The product contained curcumin, quercetin, hyaluronic acid and chondroitin sulfate (94). NIH-CPSI pain and urinary-domain scores improved significantly from baseline at 30 days, but these differences were not significant at 90 days.

In experimental systems, curcumin can inhibit NF-κB, cyclooxygenase-2 and signal transducer and activator of transcription 3 (STAT3). It can also inhibit c-Jun N-terminal kinase (JNK) while activating Nrf2-related antioxidant responses. These pathways intersect with the inflammatory-redox environment described in Section 3. In pain models outside CP/CPPS, curcumin attenuates hypersensitivity and glial inflammatory signaling, including spinal inflammasome and cytokine pathways. In the spared-nerve-injury study, curcumin was administered intraperitoneally (95, 96). Oral exposure varies with solubility, metabolism and formulation (20).

#### 4.2.2 Multi-ingredient antioxidant formulations

Several clinical studies in men with CP/CPPS have evaluated fixed combinations. A preparation containing lycopene, epigallocatechin gallate, ellagic acid, selenium and zinc was studied in inflammatory category IIIA disease. It was associated with reductions in seminal leukocytes and NIH-CPSI pain (97). *Serenoa repens* combined with selenium and lycopene, and other proprietary botanical products, have also shown symptom signals in selected cohorts (82).

In the five-component study, treated men with inflammatory category IIIA disease had fewer leukocytes in seminal fluid and expressed prostatic secretions (97). Selected semen variables and NIH-CPSI pain scores also improved relative to untreated controls. A small randomized add-on trial in 47 men examined a mineral-vitamin antioxidant complex in inflammatory category IIIA disease. Antioxidant status and symptom scores improved more than with standard therapy alone (98). Leukocyte counts in expressed prostatic secretions changed less clearly. This discordance matters when selecting biomarkers: symptoms, antioxidant status and inflammatory-cell counts need not change together.

In rat prostatitis models, lycopene has antioxidant and anti-inflammatory activity; broader phytochemical research examines Nrf2/NF-κB signaling (40, 41). These constituent effects offer possible explanations for the clinical findings. Lycopene supports lipid-phase antioxidant protection and is relevant to prostatic tissue exposure, whereas epigallocatechin gallate influences NF-κB- and Nrf2-related signaling. Selenium and zinc are nutritional co-components that contribute to antioxidant and immune functions through distinct pathways.

### 4.3 Emerging food-derived candidates

#### 4.3.1 β-Caryophyllene

This sesquiterpene occurs in black pepper, cloves and other culinary spices. Dietary intake differs from the concentrated preparations used experimentally. For this compound and the two candidates below, the reviewed literature provides experimental support but no direct human CP/CPPS intervention evidence.

Receptor studies identify β-caryophyllene as an agonist of the cannabinoid-2 receptor (99). These receptors, expressed on immune cells and glia, regulate cytokine release and neuroimmune signaling without cannabinoid-1-mediated psychotropic effects. In mouse models of neuropathic pain, β-caryophyllene reduced inflammatory signaling and pain-like behavior (100, 101). Activity was also reported in a mouse model of interstitial cystitis (102), extending the experimental rationale to an adjacent urological condition.

β-Caryophyllene is lipophilic and has been administered orally in experimental studies (99, 103). Human dose-exposure measurements would help connect the experimental findings with formulation and dose selection for clinical testing.

#### 4.3.2 Berberine

Derived from Coptis and Berberis species, this isoquinoline alkaloid is consumed mainly as a concentrated supplement rather than as a habitual dietary constituent. Its relevance here centers on gut-luminal and microbiota-mediated exposure.

Experimental studies report anti-inflammatory and antioxidant activity alongside changes in intestinal microbial composition, barrier-related signaling and microbial metabolite production. Berberine also influences NF-κB, MAPK and AMP-activated protein kinase pathways. These findings suggest possible links between microbial metabolites, systemic immune regulation and neuroendocrine signaling within a proposed gut-brain-prostate axis (104, 105). In rats with prostatitis induced by complete Freund’s adjuvant, berberine reduced prostatic inflammation and oxidative stress (104). A separate mouse model of depression-like behavior showed reduced microglial activation and NLRP3-related neuroinflammation (106).

Its low systemic oral bioavailability shifts attention toward gut-luminal actions and microbial or circulating metabolites (23, 107, 108). To examine their relevance in patients, studies should pair a characterized oral formulation with microbial function or metabolites, host inflammatory responses and pain outcomes. Antibiotics, diet and bowel function should be recorded as potential modifiers (23, 104, 107).

#### 4.3.3 Sulforaphane

This food-derived isothiocyanate forms from glucoraphanin when cruciferous vegetables are chopped or chewed. Exposure assessment should account for the food matrix, myrosinase activity and microbial conversion.

In a 2025 experimental autoimmune prostatitis study, treated mice showed less prostatic inflammatory infiltration and reduced mechanical pain responses. Animal and cell experiments implicated increased Nrf2/heme oxygenase-1 signaling and suppressed NLRP3 inflammasome activation (109). A broader genitourinary review summarizes isothiocyanates across preclinical and clinical settings (25). These findings identify peripheral redox-inflammasome signaling as a candidate pathway for translational CP/CPPS research.

### 4.4 Integrating clinical evidence with pain-sensitization biology

Clinical trials support symptom benefits from several plant-derived preparations. Curcumin and antioxidant studies contribute formulation-level evidence. Across these studies, outcomes include pain and multidomain symptom scores, urinary function and peripheral inflammatory markers (71, 81, 88, 93, 97).

The experimental literature links these clinical observations to several candidate pathways. A sulforaphane study in an experimental prostatitis model implicates peripheral redox and inflammasome signaling. β-Caryophyllene and berberine studies in interstitial cystitis, prostatitis and depression-like behavior models inform neuroimmune and microbiota-related hypotheses (99, 102, 104, 106). Quercetin and curcumin have additional mechanistic support from experimental pain models, including studies using non-oral administration. Together, these findings offer testable hypotheses for spinal and supraspinal modulation in CP/CPPS.

The pain-sensitization framework brings these findings together through a model of peripheral input modulation (Figure 3). Selected preparations may reduce the inflammatory, oxidative, urinary or nociceptive signals that sustain symptoms in particular CP/CPPS phenotypes. Reduced afferent input could, in turn, lessen spinal and supraspinal pain amplification. This proposed link can be examined by pairing clinical outcomes with measures of exposure, peripheral biology and pain processing.

**Figure 3:**
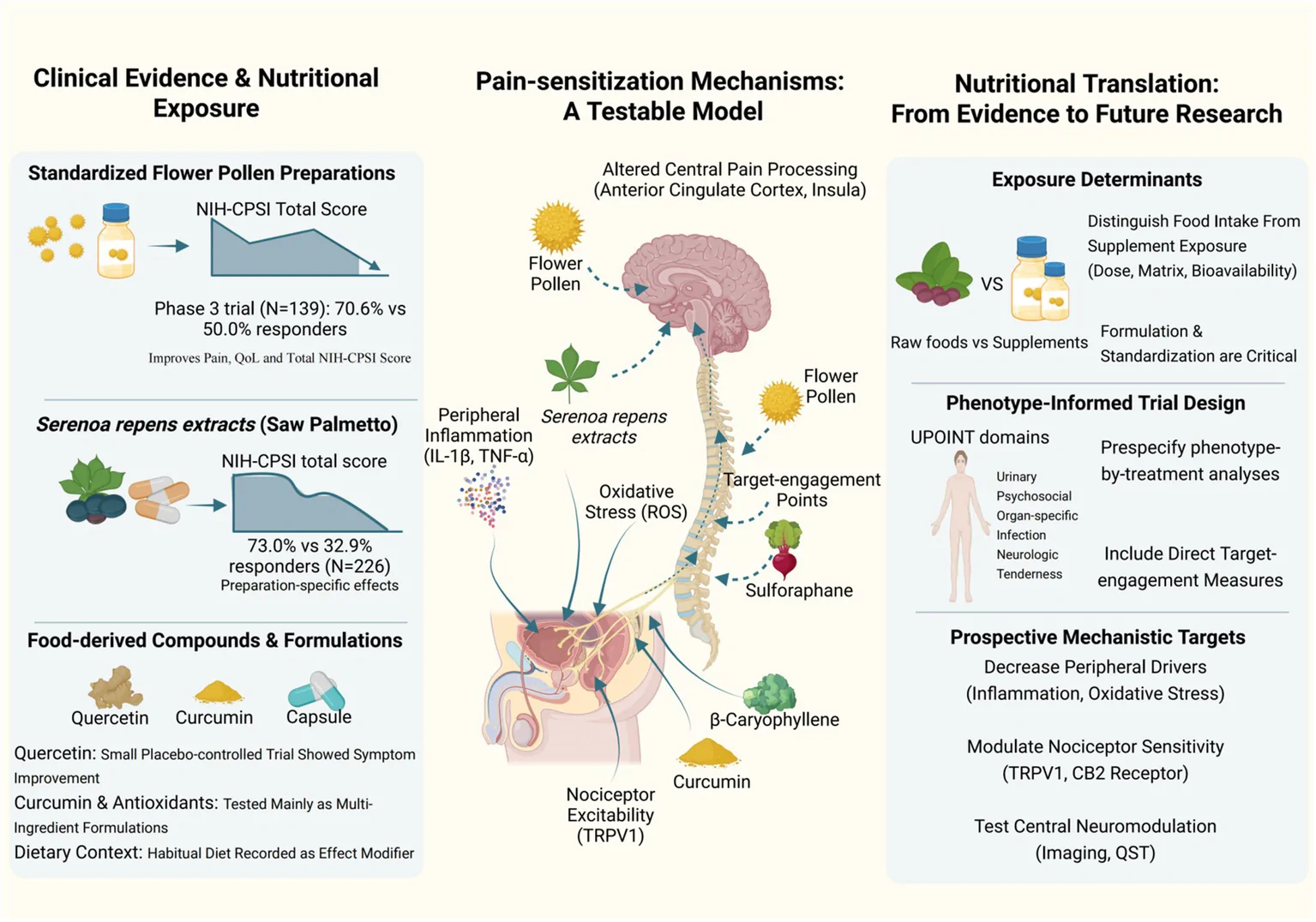
Human CP/CPPS evidence for the main plant-derived exposures (left), proposed sites of action (centre) and the resulting research agenda (right). Dashed arrows indicate hypothesized sites of action for prospective testing. QST, quantitative sensory testing (the figure was created with BioRender).

## 5 Nutritional translation and future research

### 5.1 Evidence limitations and inconsistency

The clinical literature varies in depth. Small samples and short follow-up limit precision and assessment of durability, and single-center recruitment can restrict generalizability. Incomplete reporting of allocation concealment, blinding or missing outcomes complicates bias assessment; formulations, background therapies and outcome definitions also vary (7, 75). Total NIH-CPSI scores often combine pain, urinary symptoms and quality of life, which can hide the domain responsible for improvement. Symptom fluctuation, placebo response and regression to the mean are particularly relevant in uncontrolled or short studies (110). Patient selection adds inconsistency. Category IIIA and IIIB disease, urinary-predominant presentations, pelvic-floor tenderness and widespread-pain phenotypes are often combined within one cohort (18, 57). A locally anti-inflammatory preparation may show little average benefit in an unselected population even if an inflammatory subgroup responds. *Post hoc* subgroup findings, however, can create false precision when the study was not designed to test them.

Commercial involvement differed between major trials: the Cernilton study disclosed sponsor contributions to design, conduct and data collection, whereas the *Serenoa repens* study reported company-supplied medication but no funder role in design, analysis or publication decisions (71, 81). Funding and conflict-of-interest disclosures should therefore be appraised alongside trial methods; commercial involvement alone does not establish biased results.

Positive findings from individual trials can coexist with uncertainty in broader evidence syntheses. Trials suggest benefits from selected pollen, quercetin and *Serenoa repens* preparations, while broader reviews reflect variation in the interventions and populations assessed (7, 75). In the Cochrane review, the short-term total NIH-CPSI meta-analysis included five randomized trials and 320 participants and suggested a mean reduction of 5.02 points. Certainty was low because of study limitations, inconsistency and imprecision (7). The broader phytotherapy evidence set comprised seven studies and 551 participants and combined different interventions. These pooled findings describe the overall evidence base, while individual trials clarify the effects of the preparations tested. Separate domain scores and responder thresholds would make the clinical meaning clearer.

A 2025 meta-analysis reported a non-significant NIH-CPSI estimate with very low certainty for a subgroup labeled “pollen extracts.” However, the listed studies included quercetin and curcumin–Calendula interventions (111). This mixed grouping limits pollen-specific interpretation and does not provide a like-for-like comparison with the standardized-pollen phase 3 trial. The 2025 American Urological Association guideline conditionally recommends that clinicians may prescribe selected phytotherapeutics, including saw palmetto, quercetin and pollen extract, to improve pain, voiding symptoms and quality of life (8). These recommendations provide a clinical context for evaluating the preparations with supportive trial evidence.

Each trial design contributes different information. Active-comparator studies reveal relative domain effects, placebo-controlled trials estimate product-specific efficacy, and add-on studies evaluate benefit within background care. Uncontrolled cohorts are useful for feasibility and durability signals. Interpretation should match the question each design can answer, especially when studies examine many questionnaire domains, biomarkers and time points. The clinical and mechanistic literatures offer complementary perspectives. CP/CPPS trials mainly assess symptoms or peripheral inflammatory markers, whereas central mechanisms come largely from neuropathic, inflammatory or central nervous system models. Prospective trials that combine these approaches could explain how changes in peripheral biology and pain processing relate to symptom improvement.

This review combined searches of PubMed, Web of Science Core Collection and Scopus with purposive selection; it did not include duplicate screening or formal risk-of-bias assessment. The reporting of the review’s aims and literature search was informed by items 2 and 3, respectively, of the Scale for the Assessment of Narrative Review Articles (SANRA) (112). This structured narrative synthesis integrates clinical findings with experimental mechanisms to develop a framework for nutritional translation.

### 5.2 From foods to standardized exposure: identity, bioavailability and safety

A food-derived molecule and a dietary intervention are distinct exposure categories. Habitual intake, purified compounds, standardized extracts and proprietary combinations differ in dose, matrix, pharmacokinetics and regulatory oversight. These features shape exposure and help interpret clinical and nutritional findings. Curcumin has low, formulation-dependent bioavailability; quercetin undergoes extensive intestinal and hepatic conjugation; and berberine is poorly absorbed (20, 21, 23, 113). Parenteral and intrathecal experiments identify pathway activity under controlled exposure conditions (20, 95). Nutritional translation therefore involves relating the formulation and route of administration to achievable exposure and observed clinical outcomes.

Exposure should be documented at three levels. The product needs a reproducible chemical profile and dose. The participant needs adherence data and, where feasible, measurement of circulating parent compounds or metabolites. The target needs a plausible temporal and concentration relationship to that exposure. This chain helps distinguish inadequate delivery from biological inactivity and can identify the active constituent or metabolite. It is especially relevant to quercetin conjugates, curcumin formulations and berberine metabolites, whose activities may differ from parent compounds used in cell experiments (20, 21, 107). Human quercetin studies make the exposure problem concrete. A systematic review and meta-analysis of 31 interventions found large differences in bioavailability according to glycoside form, encapsulation and food matrix; dietary fat and fiber also modified exposure (113). Lycopene absorption similarly depends on isomerization, stability, micelle formation, food composition and delivery system (114). Product comparisons and food-supplement translation therefore require more than the nominal milligram dose.

Botanical extracts also vary in active-constituent content, extraction method and batch consistency (22, 115). Defined marker compounds and transparent manufacturing information are needed if findings are to be reproduced across studies. This issue is particularly important for pollen extracts, *Serenoa repens* and multi-ingredient formulations, for which nominally similar products may not be chemically equivalent. Food matrices create biological and interpretive differences. Foods provide repeated low-dose exposures with fiber, lipids, micronutrients and other phytochemicals that alter absorption and microbial transformation (20–23, 114). Supplements may achieve higher exposures but introduce formulation and manufacturing variables. A quercetin capsule, curcumin suppository and lipidosterolic Serenoa extract each represent a different exposure from their source foods. Nutritional interpretation therefore rests on exposure biology.

Short-term trials and safety reviews generally describe the studied products as reasonably well tolerated, with gastrointestinal symptoms among the more common adverse effects (23, 116, 117). Long-term and high-dose safety is less defined. Quercetin and berberine may alter exposure to concomitant medicines. Rare cases of acute liver injury have been reported with some turmeric-containing supplements, including formulations designed to enhance bioavailability (23, 117, 118). Multi-ingredient products also require attention to constituent and drug interactions. Documenting supplement use is especially important in patients receiving several treatments.

### 5.3 Phenotype-informed nutritional trials

CP/CPPS heterogeneity favors a phenotype-informed supplementation strategy. UPOINT distinguishes urinary, psychosocial, organ-specific, infection, neurologic/systemic and tenderness domains and can support interpretation (18, 65, 119). Clinical studies of *Serenoa repens* and pollen have reported pain, urinary and quality-of-life outcomes, while experimental studies of quercetin, curcumin, β-caryophyllene and berberine provide neuroimmune insights. Together, these findings provide a basis for prospective phenotype-by-treatment hypotheses. Nutritional adjuncts fit naturally within multimodal care. Pelvic-floor tenderness, psychological distress and sexual dysfunction may require pelvic-floor, psychological, pain or sexual-medicine interventions (5, 8). Plant-derived interventions can then be evaluated as complements to domain-specific care.

Future trials should test chemically characterized products in clearly defined populations against a specified background regimen. The NIH-CPSI pain domain is a suitable primary clinical outcome; urinary, quality-of-life and global-response outcomes should be reported separately (120, 121). Trials that examine central mechanisms can select focused psychophysical, symptom, imaging or biological measures (12, 33, 52, 57). A 2025 single-arm acupuncture study paired NIH-CPSI change with pre/post structural MRI in 25 patients, showing that longitudinal central endpoints are feasible in CP/CPPS (122). Randomized designs can build on this feasibility while keeping patient-important pain outcomes primary. Follow-up should be long enough to distinguish transient symptom fluctuation from sustained benefit. Trials of multi-ingredient products should disclose their full composition and dose. Where feasible, comparisons should test whether the combination adds value beyond its principal component or background care.

A practical trial would use a prospective run-in period to characterize baseline symptom variability before randomization (110). Phenotype strata should be prespecified. The primary endpoint should emphasize the NIH-CPSI pain domain or another defined pain measure, with total score, urinary symptoms, quality of life and global response reported separately (120, 121). Responder thresholds should be prespecified and accompanied by continuous effects because their behavior depends on baseline severity. This approach accords with recent MAPP recommendations for precision trials. These emphasize explicit eligibility across widespread-pain, chronic-overlapping-pain and pelvic-floor phenotypes, mechanism-relevant treatment selection, separate pain and urinary outcomes, and adaptive designs where appropriate (123). A systematic review found that current male chronic-pelvic-pain classification systems are generally of low-to-moderate quality (119). Prospective subgroup hypotheses can improve phenotyping while these systems continue to mature.

Early response data illustrate the potential of phenotype-by-treatment interactions. In an exploratory observational cohort of 40 patients with urological chronic pelvic pain, widespread pain appeared to modify response to centrally directed and peripheral therapies (124). Although the subgroups were *post hoc* and treatment was not randomized, the pattern provides a hypothesis that future nutraceutical trials could prespecify. Mechanistic evaluation can relate exposure, peripheral biology and pain processing through measurements taken at corresponding time points. The patterns of change in these measures and patient-reported pain can help refine the proposed pathways and guide formal mediation analyses.

Pain-processing measures can be selected to match the proposed mechanism. Quantitative sensory testing can assess local and remote pressure sensitivity, temporal summation and endogenous pain inhibition. Symptom instruments can characterize widespread or nociplastic features, and neuroimaging can test prespecified pain-related networks (12, 33, 52, 57). These measures can complement a patient-important pain endpoint. Habitual diet should be recorded as a potential effect modifier when concentrated botanical preparations are evaluated (17, 66). Current studies focus mainly on extracts and supplements. Whole-food, functional-food, precision-nutrition and microbiota-informed strategies therefore represent a largely open research space in CP/CPPS.

## 6 Conclusion

Selected plant-derived bioactives and nutraceuticals have shown symptom benefits in male CP/CPPS, with direct clinical evidence for standardized pollen preparations and defined *Serenoa repens* extracts and preliminary evidence for quercetin from a small placebo-controlled trial. Curcumin and antioxidant products have mainly been evaluated as fixed combinations. Together, clinical and experimental findings support a working model of peripheral input modulation centered on inflammation, oxidative stress, nociceptor excitability and lower urinary tract function. Reduced afferent input may, in turn, lessen spinal and supraspinal pain amplification. This framework connects the clinical findings with pain-sensitization biology and highlights formulation, biological exposure and patient phenotype as priorities for nutritional translation. Given the preparation-specific and heterogeneous evidence, larger, well-designed randomized controlled trials are needed before broad clinical recommendations can be made.

## References

[1] Krieger JN Nyberg L Nickel JC. NIH consensus definition and classification of prostatitis. JAMA. (1999) 282:236–7. doi: 10.1001/jama.282.3.236, 10422990

[2] Habermacher GM Chason JT Schaeffer AJ. Prostatitis/chronic pelvic pain syndrome. Annu Rev Med. (2006) 57:195–206. doi: 10.1146/annurev.med.57.011205.135654, 16409145

[3] Krieger JN Ross SO Riley DE. Chronic prostatitis: epidemiology and role of infection. Urology. (2002) 60:8–12; discussion 13. doi: 10.1016/s0090-4295(02)02294-x, 12521579

[4] Borgert BJ Wallen EM Pham MN. Prostatitis: a review. JAMA. (2025) 334:1003–13. doi: 10.1001/jama.2025.11499, 40788632

[5] Rees J Abrahams M Doble A Cooper A, Prostatitis Expert Reference Group (Perg). Diagnosis and treatment of chronic bacterial prostatitis and chronic prostatitis/chronic pelvic pain syndrome: a consensus guideline. BJU Int. (2015) 116:509–25. doi: 10.1111/bju.13101, 25711488 PMC5008168

[6] Alshahrani S Fathi BA Abouelgreed TA El-Metwally A. Prevalence of sexual dysfunction with chronic prostatitis/chronic pelvic pain syndrome (CP/CPPS): an updated systematic review and meta-analysis. Medicina (Kaunas). (2025) 61:1110. doi: 10.3390/medicina61061110, 40572798 PMC12195409

[7] Franco JV Turk T Jung JH Xiao YT Iakhno S Tirapegui FI . Pharmacological interventions for treating chronic prostatitis/chronic pelvic pain syndrome. Cochrane Database Syst Rev. (2019) 10:CD012552. doi: 10.1002/14651858.CD012552.pub2, 31587256 PMC6778620

[8] Lai HH Pontari MA Argoff CE Bresler L Breyer BN Chou R . Male chronic pelvic pain: AUA guideline: part II treatment of chronic prostatitis/chronic pelvic pain syndrome. J Urol. (2025) 214:127–37. doi: 10.1097/JU.0000000000004565, 40243102

[9] Lai HH Pontari MA Argoff CE Bresler L Breyer BN Chou R . Male chronic pelvic pain: AUA guideline: part I evaluation and management approach. J Urol. (2025) 214:116–26. doi: 10.1097/ju.0000000000004564, 40243110

[10] Breser ML Salazar FC Rivero VE Motrich RD. Immunological mechanisms underlying chronic pelvic pain and prostate inflammation in chronic pelvic pain syndrome. Front Immunol. (2017) 8:898. doi: 10.3389/fimmu.2017.00898, 28824626 PMC5535188

[11] Wong L Done JD Schaeffer AJ Thumbikat P. Experimental autoimmune prostatitis induces microglial activation in the spinal cord. Prostate. (2015) 75:50–9. doi: 10.1002/pros.22891, 25263093 PMC4257869

[12] Farmer MA Chanda ML Parks EL Baliki MN Apkarian AV Schaeffer AJ. Brain functional and anatomical changes in chronic prostatitis/chronic pelvic pain syndrome. J Urol. (2011) 186:117–24. doi: 10.1016/j.juro.2011.03.027, 21571326 PMC4889821

[13] He H Luo H Qian B Xu H Zhang G Zou X . Autonomic nervous system dysfunction is related to chronic prostatitis/chronic pelvic pain syndrome. World J Mens Health. (2024) 42:1–28. doi: 10.5534/wjmh.220248, 37118962 PMC10782122

[14] Yilmaz U Liu YW Berger RE Yang CC. Autonomic nervous system changes in men with chronic pelvic pain syndrome. J Urol. (2007) 177:2170–4. doi: 10.1016/j.juro.2007.01.144, 17509311

[15] Anderson RU Orenberg EK Chan CA Morey A Flores V. Psychometric profiles and hypothalamic-pituitary-adrenal axis function in men with chronic prostatitis/chronic pelvic pain syndrome. J Urol. (2008) 179:956–60. doi: 10.1016/j.juro.2007.10.084, 18207189 PMC2694575

[16] Huang X Qin Z Cui H Chen J Liu T Zhu Y . Psychological factors and pain catastrophizing in men with chronic prostatitis/chronic pelvic pain syndrome (cp/Cpps): a meta-analysis. Transl Androl Urol. (2020) 9:485–93. doi: 10.21037/tau.2020.01.25, 32420154 PMC7214995

[17] Hashemi N Tondro Anamag F Javan Balegh Marand A Rahnama’I MS Herizchi Ghadim H Salehi-Pourmehr H . A systematic and comprehensive review of the role of microbiota in urinary chronic pelvic pain syndrome. Neurourol Urodyn. (2024) 43:1859–82. doi: 10.1002/nau.25550, 38994675

[18] Shoskes DA Nickel JC Dolinga R Prots D. Clinical phenotyping of patients with chronic prostatitis/chronic pelvic pain syndrome and correlation with symptom severity. Urology. (2009) 73:538–42. doi: 10.1016/j.urology.2008.09.074, 19118880

[19] Dashdondov O Wazir J Sukhbaatar G Mikrani R Dorjsuren B Aktar N . Herbal nutraceutical treatment of chronic prostatitis-chronic pelvic pain syndrome: a literature review. Int Urol Nephrol. (2021) 53:1515–28. doi: 10.1007/s11255-021-02868-w, 33907984

[20] Jamwal R. Bioavailable curcumin formulations: a review of pharmacokinetic studies in healthy volunteers. J Integr Med. (2018) 16:367–74. doi: 10.1016/j.joim.2018.07.001, 30006023

[21] Almeida AF Borge GIA Piskula M Tudose A Tudoreanu L Valentová K . Bioavailability of quercetin in humans with a focus on interindividual variation. Compr Rev Food Sci Food Saf. (2018) 17:714–31. doi: 10.1111/1541-4337.12342, 33350133

[22] Marti G Joulia P Amiel A Fabre B David B Fabre N . Comparison of the phytochemical composition of *Serenoa repens* extracts by a multiplexed metabolomic approach. Molecules (Basel, Switzerland). (2019) 24:2208. doi: 10.3390/molecules24122208, 31200456 PMC6631273

[23] Khoshandam A Imenshahidi M Hosseinzadeh H. Pharmacokinetic of berberine, the main constituent of *Berberis vulgaris* L.: a comprehensive review. Phytother Res. (2022) 36:4063–79. doi: 10.1002/ptr.7589, 36221815

[24] Doiron RC Shoskes DA Nickel JC. Male cp/Cpps: where do we stand. World J Urol. (2019) 37:1015–22. doi: 10.1007/s00345-019-02718-6, 30864007

[25] Piragine E Moretti P Scarpulla S Calderone V Martelli A. Potential efficacy of polyphenols and isothiocyanates in the management of genitourinary diseases: a systematic review of preclinical and clinical studies. Int J Mol Sci. (2026) 27:1660. doi: 10.3390/ijms27041660, 41751796 PMC12940174

[26] Shao B Wu K Fan Z Gao X Wan S Xiao L . Current research hotspots and difficulties of chronic prostatitis/chronic pelvic pain syndrome: neuroendocrine mechanism. Ann Med. (2026) 58:2616886. doi: 10.1080/07853890.2026.2616886, 41555741 PMC12821347

[27] Latremoliere A Woolf CJ. Central sensitization: a generator of pain hypersensitivity by central neural plasticity. J Pain. (2009) 10:895–926. doi: 10.1016/j.jpain.2009.06.012, 19712899 PMC2750819

[28] Paulis G Conti E Voliani S Bertozzi MA Sarteschi ML Menchini Fabris F. Evaluation of the cytokines in genital secretions of patients with chronic prostatitis. Arch Ital Urol Androl. (2003) 75:179–86. 15005490

[29] Desireddi NV Campbell PL Stern JA Sobkoviak R Chuai S Shahrara S . Monocyte chemoattractant protein-1 and macrophage inflammatory protein-1alpha as possible biomarkers for the chronic pelvic pain syndrome. J Urol. (2008) 179:1857–61. doi: 10.1016/j.juro.2008.01.028, 18353390 PMC2786778

[30] Lundh D Hedelin H Jonsson K Gifford M Larsson D. Assessing chronic pelvic pain syndrome patients: blood plasma factors and cortisol saliva. Scand J Urol. (2013) 47:521–8. doi: 10.3109/21681805.2013.769460, 23394140

[31] Chen L Bian Z Chen J Meng J Zhang M Liang C. Immunological alterations in patients with chronic prostatitis/chronic pelvic pain syndrome and experimental autoimmune prostatitis model: a systematic review and meta-analysis. Cytokine. (2021) 141:155440. doi: 10.1016/j.cyto.2021.155440, 33550164

[32] Davis SNP Maykut CA Binik YM Amsel R Carrier S. Tenderness as measured by pressure pain thresholds extends beyond the pelvis in chronic pelvic pain syndrome in men. J Sex Med. (2011) 8:232–9. doi: 10.1111/j.1743-6109.2010.02041.x, 20946176

[33] Harte SE Schrepf A Gallop R Kruger GH Lai HHH Sutcliffe S . Quantitative assessment of nonpelvic pressure pain sensitivity in urologic chronic pelvic pain syndrome: a Mapp research network study. Pain. (2019) 160:1270–80. doi: 10.1097/j.pain.0000000000001505, 31050659 PMC6527452

[34] Korkmaz S Karadag MA Hamamcioglu K Sofikerim M Aksu M. Electrophysiological identification of central sensitization in patients with chronic prostatitis. Urol J. (2015) 12:2280–4. 26341773

[35] Zhao Y Lin J Dong Y Tian Z Ye Y Ma Z . Neuroimaging studies of chronic prostatitis/chronic pelvic pain syndrome. Pain Res Manag. (2022) 2022:9448620. doi: 10.1155/2022/9448620, 35573644 PMC9095382

[36] Done JD Rudick CN Quick ML Schaeffer AJ Thumbikat P. Role of mast cells in male chronic pelvic pain. J Urol. (2012) 187:1473–82. doi: 10.1016/j.juro.2011.11.116, 22341813 PMC3662223

[37] Potts JM Pasqualotto FF. Seminal oxidative stress in patients with chronic prostatitis. Andrologia. (2003) 35:304–8. doi: 10.1046/j.1439-0272.2003.00572.x, 14535860

[38] Kullisaar T Türk S Punab M Korrovits P Kisand K Rehema A . Oxidative stress in leucocytospermic prostatitis patients: preliminary results. Andrologia. (2008) 40:161–72. doi: 10.1111/j.1439-0272.2007.00816.x, 18477203

[39] Paulis G. Inflammatory mechanisms and oxidative stress in prostatitis: the possible role of antioxidant therapy. Res Rep Urol. (2018) 10:75–87. doi: 10.2147/rru.s170400, 30271757 PMC6149977

[40] Zhao Q Yang F Meng L Chen D Wang M Lu X . Lycopene attenuates chronic prostatitis/chronic pelvic pain syndrome by inhibiting oxidative stress and inflammation via the interaction of Nf-κB, Mapks, and Nrf2 signaling pathways in rats. Andrology. (2020) 8:747–55. doi: 10.1111/andr.12747, 31880092 PMC7317562

[41] Wu S Liao X Zhu Z Huang R Chen M Huang A . Antioxidant and anti-inflammation effects of dietary phytochemicals: the Nrf2/Nf-κB signalling pathway and upstream factors of Nrf2. Phytochemistry. (2022) 204:113429. doi: 10.1016/j.phytochem.2022.113429, 36096269

[42] Zhang F Zhang A Meng T Liu X Yang C Liang C . Epithelial redox stress programs macrophage immunometabolism through a Znf24-Mif-Nf-κB pathway in chronic nonbacterial prostatitis. Redox Biol. (2026) 90:104042. doi: 10.1016/j.redox.2026.104042, 41576699 PMC12859805

[43] Miller LJ Fischer KA Goralnick SJ Litt M Burleson JA Albertsen P . Nerve growth factor and chronic prostatitis/chronic pelvic pain syndrome. Urology. (2002) 59:603–8. doi: 10.1016/s0090-4295(01)01597-7, 11927336

[44] Roman K Hall C Schaeffer AJ Thumbikat P. Trpv1 in experimental autoimmune prostatitis. Prostate. (2020) 80:28–37. doi: 10.1002/pros.23913, 31573117 PMC7313375

[45] Aydogdu O Gocun PU Aronsson P Carlsson T Winder M. Cross-organ sensitization between the prostate and bladder in an experimental rat model of lipopolysaccharide (LPS)-induced chronic pelvic pain syndrome. BMC Urol. (2021) 21:113. doi: 10.1186/s12894-021-00882-9, 34419040 PMC8380371

[46] Funahashi Y Takahashi R Mizoguchi S Suzuki T Takaoka E Ni J . Bladder overactivity and afferent hyperexcitability induced by prostate-to-bladder cross-sensitization in rats with prostatic inflammation. J Physiol. (2019) 597:2063–78. doi: 10.1113/jp277452, 30666643 PMC6441933

[47] Hetrick DC Glazer H Liu YW Turner JA Frest M Berger RE. Pelvic floor electromyography in men with chronic pelvic pain syndrome: a case-control study. Neurourol Urodyn. (2006) 25:46–9. doi: 10.1002/nau.20162, 16167354

[48] Shoskes DA Berger R Elmi A Landis JR Propert KJ Zeitlin S . Muscle tenderness in men with chronic prostatitis/chronic pelvic pain syndrome: the chronic prostatitis cohort study. J Urol. (2008) 179:556–60. doi: 10.1016/j.juro.2007.09.088, 18082223 PMC2664648

[49] Yani MS Eckel SP Kirages DJ Rodriguez LV Corcos DM Kutch JJ. Impaired ability to relax pelvic floor muscles in men with chronic prostatitis/chronic pelvic pain syndrome. Phys Ther. (2022) 102:pzac059. doi: 10.1093/ptj/pzac059, 35576002 PMC9618172

[50] Van Reijn-Baggen DA Han-Geurts IJM Voorham-Van Der Zalm PJ Pelger RCM Hagenaars-Van Miert CHAC Laan ETM. Pelvic floor physical therapy for pelvic floor hypertonicity: a systematic review of treatment efficacy. Sex Med Rev. (2022) 10:209–30. doi: 10.1016/j.sxmr.2021.03.002, 34127429

[51] Ji RR Nackley A Huh Y Terrando N Maixner W. Neuroinflammation and central sensitization in chronic and widespread pain. Anesthesiology. (2018) 129:343–66. doi: 10.1097/ALN.0000000000002130, 29462012 PMC6051899

[52] Kutch JJ Yani MS Asavasopon S Kirages DJ Rana M Cosand L . Altered resting state neuromotor connectivity in men with chronic prostatitis/chronic pelvic pain syndrome: a Mapp: research network neuroimaging study. Neuroimage Clin. (2015) 8:493–502. doi: 10.1016/j.nicl.2015.05.013, 26106574 PMC4474411

[53] Woodworth D Mayer E Leu K Ashe-Mcnalley C Naliboff BD Labus JS . Unique microstructural changes in the brain associated with urological chronic pelvic pain syndrome (Ucpps) revealed by diffusion tensor Mri, super-resolution track density imaging, and statistical parameter mapping: a Mapp network neuroimaging study. PLoS One. (2015) 10:e0140250. doi: 10.1371/journal.pone.0140250, 26460744 PMC4604194

[54] Wang C Kutch JJ Labus JS Yang CC Harris RE Mayer EA . Reproducible microstructural changes in the brain associated with the presence and severity of urologic chronic pelvic pain syndrome (Ucpps): a 3-year longitudinal diffusion tensor imaging study from the Mapp network. J Pain. (2023) 24:627–42. doi: 10.1016/j.jpain.2022.11.008, 36435486 PMC10676766

[55] Ge S Xiang Y Hua X Wang Z Hu Q Guo Y . The characteristics of brain function alterations in patients with chronic prostatitis/chronic pelvic pain syndrome across varying symptom severities evaluated by Nih-Cpsi. Front Neurosci. (2025) 19:1511654. doi: 10.3389/fnins.2025.1511654, 40078709 PMC11897570

[56] Kosek E. The concept of nociplastic pain-where to from here? Pain. (2024) 165:S50–7. doi: 10.1097/j.pain.0000000000003305, 39560415

[57] Schrepf A Gallop R Naliboff B Harte SE Afari N Lai HH . Clinical phenotyping for pain mechanisms in urologic chronic pelvic pain syndromes: a Mapp research network study. J Pain. (2022) 23:1594–603. doi: 10.1016/j.jpain.2022.03.240, 35472518 PMC10547025

[58] Krsmanovic A Tripp DA Nickel JC Shoskes DA Pontari M Litwin MS . Psychosocial mechanisms of the pain and quality of life relationship for chronic prostatitis/chronic pelvic pain syndrome (CP/CPPS). Can Urol Assoc J. (2014) 8:403–8. doi: 10.5489/cuaj.2179, 25553153 PMC4277519

[59] Schrepf A Naliboff B Williams DA Stephens-Shields AJ Landis JR Gupta A . Adverse childhood experiences and symptoms of urologic chronic pelvic pain syndrome: a multidisciplinary approach to the study of chronic pelvic pain research network study. Ann Behav Med. (2018) 52:865–77. doi: 10.1093/abm/kax060, 30212850 PMC6135957

[60] Riegel B Bruenahl CA Ahyai S Bingel U Fisch M Löwe B. Assessing psychological factors, social aspects and psychiatric co-morbidity associated with chronic prostatitis/chronic pelvic pain syndrome (cp/Cpps) in men -- a systematic review. J Psychosom Res. (2014) 77:333–50. doi: 10.1016/j.jpsychores.2014.09.012, 25300538

[61] Bai J Gu L Chen Y Liu X Yang J Li M . Evaluation of psychological stress, cortisol awakening response, and heart rate variability in patients with chronic prostatitis/chronic pelvic pain syndrome complicated by lower urinary tract symptoms and erectile dysfunction. Front Psychol. (2022) 13:903250. doi: 10.3389/fpsyg.2022.903250, 36405196 PMC9674019

[62] Arora HC Eng C Shoskes DA. Gut microbiome and chronic prostatitis/chronic pelvic pain syndrome. Ann Transl Med. (2017) 5:30. doi: 10.21037/atm.2016.12.32, 28217695 PMC5300850

[63] Wang S Zang M Yang X Lv L Chen L Cui J . Gut microbiome in men with chronic prostatitis/chronic pelvic pain syndrome: profiling and its predictive significance. World J Urol. (2023) 41:3019–26. doi: 10.1007/s00345-023-04587-6, 37684401

[64] Song S Zhang C Zhang B Yin J Yu C Wang X . Targeting the brain-gut-prostate axis in chronic prostatitis: mechanisms and therapeutics. Front Endocrinol. (2025) 16:1628094. doi: 10.3389/fendo.2025.1628094, 40687578 PMC12270845

[65] Shoskes DA Nickel JC Kattan MW. Phenotypically directed multimodal therapy for chronic prostatitis/chronic pelvic pain syndrome: a prospective study using Upoint. Urology. (2010) 75:1249–53. doi: 10.1016/j.urology.2010.01.021, 20363491

[66] Herati AS Shorter B Srinivasan AK Tai J Seideman C Lesser M . Effects of foods and beverages on the symptoms of chronic prostatitis/chronic pelvic pain syndrome. Urology. (2013) 82:1376–80. doi: 10.1016/j.urology.2013.07.015, 23978369

[67] Wang Z Wu W Wen B Xu Z Xi J Zhang Y. Dietary patterns and chronic prostatitis: a symptom severity prediction model based on nutritional clustering and machine learning. Front Nutr. (2026) 12:1660430. doi: 10.3389/fnut.2025.1660430, 41635905 PMC12863200

[68] Wagenlehner FME Bschleipfer T Pilatz A Weidner W. Pollen extract for chronic prostatitis-chronic pelvic pain syndrome. Urol Clin North Am. (2011) 38:285–92. doi: 10.1016/j.ucl.2011.04.004, 21798390

[69] Kamijo T Sato S Kitamura T. Effect of cernitin pollen-extract on experimental nonbacterial prostatitis in rats. Prostate. (2001) 49:122–31. doi: 10.1002/pros.1126, 11582591

[70] Asakawa K Nandachi N Satoh S Honma M Namikata S Ishii M . Effects of cernitin pollen-extract (Cernilton) on inflammatory cytokines in sex-hormone-induced nonbacterial prostatitis rats. Hinyokika Kiyo. (2001) 47:459–65. 11523128

[71] Wagenlehner FME Schneider H Ludwig M Schnitker J Brähler E Weidner W. A pollen extract (Cernilton) in patients with inflammatory chronic prostatitis-chronic pelvic pain syndrome: a multicentre, randomised, prospective, double-blind, placebo-controlled phase 3 study. Eur Urol. (2009) 56:544–51. doi: 10.1016/j.eururo.2009.05.046, 19524353

[72] Iwamura H Koie T Soma O Matsumoto T Imai A Hatakeyama S . Eviprostat has an identical effect compared to pollen extract (Cernilton) in patients with chronic prostatitis/chronic pelvic pain syndrome: a randomized, prospective study. BMC Urol. (2015) 15:120. doi: 10.1186/s12894-015-0115-5, 26643109 PMC4672535

[73] Matsukawa Y Naito Y Funahashi Y Ishida S Fujita T Tochigi K . Comparison of cernitin pollen extract vs tadalafil therapy for refractory chronic prostatitis/chronic pelvic pain syndrome: a randomized, prospective study. Neurourol Urodyn. (2020) 39:1994–2002. doi: 10.1002/nau.24454, 32648985

[74] Cai T Verze P La Rocca R Palmieri A Tiscione D Luciani LG . The clinical efficacy of pollen extract and vitamins on chronic prostatitis/chronic pelvic pain syndrome is linked to a decrease in the pro-inflammatory cytokine interleukin-8. World J Mens Health. (2017) 35:120–8. doi: 10.5534/wjmh.2017.35.2.120, 28497911 PMC5583369

[75] Cai T Verze P La Rocca R Anceschi U De Nunzio C Mirone V. The role of flower pollen extract in managing patients affected by chronic prostatitis/chronic pelvic pain syndrome: a comprehensive analysis of all published clinical trials. BMC Urol. (2017) 17:32. doi: 10.1186/s12894-017-0223-5, 28431537 PMC5401347

[76] Elist J. Effects of pollen extract preparation Prostat/Poltit on lower urinary tract symptoms in patients with chronic nonbacterial prostatitis/chronic pelvic pain syndrome: a randomized, double-blind, placebo-controlled study. Urology. (2006) 67:60–3. doi: 10.1016/j.urology.2005.07.035, 16413333

[77] Cai T Gallelli L Cione E Verze P Palmieri A Mirone V . The efficacy and tolerability of pollen extract in combination with hyaluronic acid and vitamins in the management of patients affected by chronic prostatitis/chronic pelvic pain syndrome: a 26 weeks, randomized, controlled, single-blinded, phase III study. Minerva Urol Nefrol. (2022) 74:780–8. doi: 10.23736/s2724-6051.21.04141-2, 33781014

[78] Macchione N Bernardini P Piacentini I Mangiarotti B Del Nero A. Flower pollen extract in association with vitamins (Deprox 500®) versus *Serenoa repens* in chronic prostatitis/chronic pelvic pain syndrome: a comparative analysis of two different treatments. Antiinflamm Antiallergy Agents Med Chem. (2019) 18:151–61. doi: 10.2174/1871523018666181128164252, 30488800 PMC6751341

[79] Maurizi A De Luca F Zanghi A Manzi E Leonardo C Guidotti M . The role of nutraceutical medications in men with non bacterial chronic prostatitis and chronic pelvic pain syndrome: a prospective non blinded study utilizing flower pollen extracts versus bioflavonoids. Arch Ital Urol Androl. (2019) 90:260–4. doi: 10.4081/aiua.2018.4.260, 30655636

[80] IvănuȚĂ M Puia D CumpănaȘ AA IvănuȚĂ AM Groza V Pricop C. Clinical evaluation of a pollen-extract-based phytotherapy compared to conventional therapies in chronic prostatitis and chronic pelvic pain syndrome. Med Sci (Basel, Switzerland). (2025) 13:186. doi: 10.3390/medsci13030186, 40981184 PMC12452326

[81] Zhang K Guo RQ Chen SW Chen B Xue XB Chen S . The efficacy and safety of *Serenoa repens* extract for the treatment of patients with chronic prostatitis/chronic pelvic pain syndrome: a multicenter, randomized, double-blind, placebo-controlled trial. World J Urol. (2021) 39:3489–95. doi: 10.1007/s00345-020-03577-2, 33452912 PMC8510895

[82] Morgia G Mucciardi G Galì A Madonia M Marchese F Di Benedetto A . Treatment of chronic prostatitis/chronic pelvic pain syndrome category IIIA with *Serenoa repens* plus selenium and lycopene (Profluss) versus *S. repens* alone: an Italian randomized multicenter-controlled study. Urol Int. (2010) 84:400–6. doi: 10.1159/000302716, 20332612

[83] Kaplan SA Volpe MA Te AE. A prospective, 1-year trial using saw palmetto versus finasteride in the treatment of category III prostatitis/chronic pelvic pain syndrome. J Urol. (2004) 171:284–8. doi: 10.1097/01.ju.0000101487.83730.80, 14665895

[84] Lok W Lin T Cao D Wei Q. Is *Serenoa repens* effective for the treatment of chronic prostatitis/chronic pelvic pain syndrome (CP/CPPS)? A systematic review and meta-analysis. Asian J Surg. (2022) 45:1746–7. doi: 10.1016/j.asjsur.2022.01.038, 35165019

[85] Lee DH Yu YD. Multi-pharmacological treatment for young subfertile males with chronic prostatitis/chronic pelvic pain syndrome. Investig Clin Urol. (2024) 65:567–78. doi: 10.4111/icu.20240191, 39505516 PMC11543650

[86] Suzuki M Oki T Sugiyama T Umegaki K Uchida S Yamada S. Muscarinic and alpha 1-adrenergic receptor binding characteristics of saw palmetto extract in rat lower urinary tract. Urology. (2007) 69:1216–20. doi: 10.1016/j.urology.2007.02.038, 17572227

[87] Chiavaroli A Recinella L Ferrante C Locatelli M Carradori S Macchione N . *Crocus sativus*, *Serenoa repens* and *Pinus massoniana* extracts modulate inflammatory response in isolated rat prostate challenged with LPS. J Biol Regul Homeost Agents. (2017) 31:531–41. 28889734

[88] Shoskes DA Zeitlin SI Shahed A Rajfer J. Quercetin in men with category III chronic prostatitis: a preliminary prospective, double-blind, placebo-controlled trial. Urology. (1999) 54:960–3. doi: 10.1016/s0090-4295(99)00358-1 10604689

[89] Meng LQ Yang FY Wang MS Shi BK Chen DX Chen D . Quercetin protects against chronic prostatitis in rat model through NF-κB and MAPK signaling pathways. Prostate. (2018) 78:790–800. doi: 10.1002/pros.23536 29654614

[90] Yang R Li L Yuan H Liu H Gong Y Zou L . Quercetin relieved diabetic neuropathic pain by inhibiting upregulated P2X4 receptor in dorsal root ganglia. J Cell Physiol. (2019) 234:2756–64. doi: 10.1002/jcp.27091 30145789

[91] Ye G Lin C Zhang Y Ma Z Chen Y Kong L . Quercetin alleviates neuropathic pain in the rat cci model by mediating Ampk/Mapk pathway. J Pain Res. (2021) 14:1289–301. doi: 10.2147/jpr.s298727, 34040433 PMC8141401

[92] Han X Xu T Fang Q Zhang H Yue L Hu G . Quercetin hinders microglial activation to alleviate neurotoxicity via the interplay between Nlrp3 inflammasome and mitophagy. Redox Biol. (2021) 44:102010. doi: 10.1016/j.redox.2021.102010, 34082381 PMC8182123

[93] Morgia G Russo GI Urzì D Privitera S Castelli T Favilla V . A phase II, randomized, single-blinded, placebo-controlled clinical trial on the efficacy of Curcumina and Calendula suppositories for the treatment of patients with chronic prostatitis/chronic pelvic pain syndrome type III. Arch Ital Urol Androl. (2017) 89:110–3. doi: 10.4081/aiua.2017.2.110, 28679180

[94] Iacovelli V Vittori M Carilli M Cipriani C Signoretti M Antonucci M . Short-term results of curcumin, quercetin, hyaluronic acid and chondroitin sulfate (Ialuril soft gels®) in the management of chronic prostatitis/primary prostate pain syndrome: a single-center prospective study. Front Surg. (2025) 12:1700681. doi: 10.3389/fsurg.2025.1700681, 41293446 PMC12640897

[95] Liu S Li Q Zhang MT Mao-Ying QL Hu LY Wu GC . Curcumin ameliorates neuropathic pain by down-regulating spinal il-1β via suppressing astroglial Nalp1 inflammasome and Jak2-Stat3 signalling. Sci Rep. (2016) 6:28956. doi: 10.1038/srep28956, 27381056 PMC4933926

[96] Hasanzadeh S Read MI Bland AR Majeed M Jamialahmadi T Sahebkar A. Curcumin: an inflammasome silencer. Pharmacol Res. (2020) 159:104921. doi: 10.1016/j.phrs.2020.104921, 32464325

[97] Lombardo F Fiducia M Lunghi R Marchetti L Palumbo A Rizzo F . Effects of a dietary supplement on chronic pelvic pain syndrome (category IIIA), leucocytospermia and semen parameters. Andrologia. (2012) 44:672–8. doi: 10.1111/j.1439-0272.2011.01248.x, 22053857

[98] Shevchenko SY Kulchavenya EV Baranchukova AA. The possibility of correcting oxidative stress in patients with inflammatory type of chronic abacterial prostatitis (chronic pelvic pain syndrome category Iiia). Urologiia. (2023) 22–9. doi: 10.18565/urology.2023.6.22-29 38156679

[99] Gertsch J Leonti M Raduner S Racz I Chen JZ Xie XQ . Beta-caryophyllene is a dietary cannabinoid. Proc Natl Acad Sci U S A. (2008) 105:9099–104. doi: 10.1073/pnas.0803601105, 18574142 PMC2449371

[100] Segat GC Manjavachi MN Matias DO Passos GF Freitas CS Costa R . Antiallodynic effect of β-caryophyllene on paclitaxel-induced peripheral neuropathy in mice. Neuropharmacology. (2017) 125:207–19. doi: 10.1016/j.neuropharm.2017.07.015, 28729222

[101] Aly E Khajah MA Masocha W. β-Caryophyllene, a CB2-receptor-selective phytocannabinoid, suppresses mechanical allodynia in a mouse model of antiretroviral-induced neuropathic pain. Molecules. (2019) 25:106. doi: 10.3390/molecules25010106, 31892132 PMC6983198

[102] Berger G Arora N Burkovskiy I Xia Y Chinnadurai A Westhofen R . Experimental cannabinoid 2 receptor activation by phyto-derived and synthetic cannabinoid ligands in LPS-induced interstitial cystitis in mice. Molecules. (2019) 24:4239. doi: 10.3390/molecules24234239, 31766439 PMC6930590

[103] Santos PS Oliveira TC R Júnior LM Figueiras A Nunes LCC. β-Caryophyllene delivery systems: enhancing the oral pharmacokinetic and stability. Curr Pharm Des. (2018) 24:3440–53. doi: 10.2174/1381612824666180912151412, 30207226

[104] Tian YQ Ren X Wang J Li X Yin YS Guo ZH . Berberine hydrochloride alleviates chronic prostatitis/chronic pelvic pain syndrome by modifying gut microbiome signaling. Asian J Androl. (2024) 26:500–9. doi: 10.4103/aja202427, 39012524 PMC11449416

[105] Kumar A Ekavali E Chopra K Mukherjee M Pottabathini R Dhull DK. Current knowledge and pharmacological profile of berberine: an update. Eur J Pharmacol. (2015) 761:288–97. doi: 10.1016/j.ejphar.2015.05.068, 26092760

[106] Qin Z Shi DD Li W Cheng D Zhang YD Zhang S . Berberine ameliorates depression-like behaviors in mice via inhibiting NLRP3 inflammasome-mediated neuroinflammation and preventing neuroplasticity disruption. J Neuroinflammation. (2023) 20:54. doi: 10.1186/s12974-023-02744-7, 36859349 PMC9976521

[107] Wang K Feng X Chai L Cao S Qiu F. The metabolism of berberine and its contribution to the pharmacological effects. Drug Metab Rev. (2017) 49:139–57. doi: 10.1080/03602532.2017.1306544, 28290706

[108] Murakami T Bodor E Bodor N. Approaching strategy to increase the oral bioavailability of berberine, a quaternary ammonium isoquinoline alkaloid: part 2. Development of oral dosage formulations. Expert Opin Drug Metab Toxicol. (2023) 19:139–48. doi: 10.1080/17425255.2023.2203858, 37060323

[109] Meng T Feng R Zhu Y Luo J Zhang A Liu Y . Sulforaphane relieved inflammation symptoms in EAP mice by blocking oxidative stress and NLRP3 inflammasome activation through the Nrf2 pathway. Clin Exp Immunol. (2025) 219:uxaf022. doi: 10.1093/cei/uxaf022, 40207573 PMC12070798

[110] Stephens-Shields AJ Clemens JQ Jemielita T Farrar J Sutcliffe S Hou X . Symptom variability and early symptom regression in the Mapp study: a prospective study of urological chronic pelvic pain syndrome. J Urol. (2016) 196:1450–5. doi: 10.1016/j.juro.2016.04.070, 27131464 PMC5069105

[111] Alshahrani S Fathi BA Abouelgreed TA El-Metwally A. Comparative efficacy of pharmacological interventions for chronic prostatitis/chronic pelvic pain syndrome: an updated systematic review and meta-analysis of randomized controlled trials. Healthcare (Basel, Switzerland). (2025) 13:2956. doi: 10.3390/healthcare13222956, 41302342 PMC12652971

[112] Baethge C Goldbeck-Wood S Mertens S. Sanra-a scale for the quality assessment of narrative review articles. Res Integr Peer Rev. (2019) 4:5. doi: 10.1186/s41073-019-0064-8, 30962953 PMC6434870

[113] Liu L Barber E Kellow NJ Williamson G. Improving quercetin bioavailability: a systematic review and meta-analysis of human intervention studies. Food Chem. (2025) 477:143630. doi: 10.1016/j.foodchem.2025.143630, 40037045

[114] Wu H Wu Y Cui Z Hu L. Nutraceutical delivery systems to improve the bioaccessibility and bioavailability of lycopene: a review. Crit Rev Food Sci Nutr. (2024) 64:6361–79. doi: 10.1080/10408398.2023.2168249, 36655428

[115] Sarma N Upton R Rose U Guo DA Marles R Khan I . Pharmacopeial standards for the quality control of botanical dietary supplements in the United States. J Diet Suppl. (2023) 20:485–504. doi: 10.1080/19390211.2021.1990171, 34699287

[116] Agbabiaka TB Pittler MH Wider B Ernst E. *Serenoa repens* (saw palmetto): a systematic review of adverse events. Drug Saf. (2009) 32:637–47. doi: 10.2165/00002018-200932080-00003, 19591529

[117] Andres S Pevny S Ziegenhagen R Bakhiya N Schäfer B Hirsch-Ernst KI . Safety aspects of the use of quercetin as a dietary supplement. Mol Nutr Food Res. (2018) 62:10.1002/mnfr.201700447. doi: 10.1002/mnfr.201700447, 29127724

[118] Lombardi N Crescioli G Maggini V Ippoliti I Menniti-Ippolito F Gallo E . Acute liver injury following turmeric use in Tuscany: an analysis of the Italian Phytovigilance database and systematic review of case reports. Br J Clin Pharmacol. (2021) 87:741–53. doi: 10.1111/bcp.14460, 32656820

[119] Cyr MP Nahon I Worman R Cowley D Hodges PW. Classification systems for chronic pelvic pain in males: a systematic review. BJU Int. (2025) 135:22–30. doi: 10.1111/bju.16485, 39075791 PMC11628891

[120] Litwin MS Mcnaughton-Collins M Fowler FJ Nickel JC Calhoun EA Pontari MA . The National Institutes of Health chronic prostatitis symptom index: development and validation of a new outcome measure. Chronic prostatitis collaborative research network. J Urol. (1999) 162:369–75. doi: 10.1016/S0022-5347(05)68562-X, 10411041

[121] Stephens-Shields AJ Lai HH Landis JR Kreder K Rodriguez LV Naliboff BD . Clinically important differences for pain and urinary symptoms in urological chronic pelvic pain syndrome: a Mapp network study. J Urol. (2023) 209:1132–40. doi: 10.1097/JU.0000000000003394, 36848118 PMC11062515

[122] Wang Z Wu J Deng C Hu Y Shi S Liu S . Efficacy and brain modulation mechanisms of acupuncture for chronic prostatitis/chronic pelvic pain syndrome revealed by structural MRI changes. Front Neurol. (2025) 16:1579484. doi: 10.3389/fneur.2025.1579484, 40635711 PMC12237666

[123] Stephens Shields AJ Clemens JQ Pontari MA Lai HH Moldwin R Williams DA . Towards precision medicine in clinical trials for the treatment of urologic chronic pelvic pain syndrome: lessons from the Mapp research network. Nat Rev Urol. (2025) 22:632–42. doi: 10.1038/s41585-025-01030-w, 40307537

[124] Schrepf A Locke K Moldwin R Williams DA Till S Farrar J . Widespread pain moderates the response to centrally-acting therapies in an observational cohort of patients with urologic chronic pelvic pain syndrome: a Mapp research network study. Neurourol Urodyn. (2025) 44:1290–5. doi: 10.1002/nau.70068, 40415481 PMC12264457

